# A Posterior Centre Establishes and Maintains Polarity of the Caenorhabditis elegans Embryo by a Wnt-Dependent Relay Mechanism

**DOI:** 10.1371/journal.pbio.0040396

**Published:** 2006-11-21

**Authors:** Marcus Bischoff, Ralf Schnabel

**Affiliations:** Technische Universität Braunschweig Carolo Wilhelmina, Institut für Genetik, Braunschweig, Germany; Cambridge University, United Kingdom

## Abstract

Cellular polarity is a general feature of animal development. However, the mechanisms that establish and maintain polarity in a field of cells or even in the whole embryo remain elusive. Here we provide evidence that in the Caenorhabditis elegans embryo, the descendants of P_1_, the posterior blastomere of the 2-cell stage, constitute a polarising centre that orients the cell divisions of most of the embryo. This polarisation depends on a MOM-2/Wnt signal originating from the P_1_ descendants. Furthermore, we show that the MOM-2/Wnt signal is transduced from cell to cell by a relay mechanism. Our findings suggest how polarity is first established and then maintained in a field of cells. According to this model, the relay mechanism constantly orients the polarity of all cells towards the polarising centre, thus organising the whole embryo. This model may also apply to other systems such as *Drosophila* and vertebrates.

## Abstract 2

In the*C. elegans* embryo, the polarizing signal that orients the cell divisions of the embryo is transduced from cell to cell by a relay mechanism.

## Introduction

Many developmental processes depend on polarised cells; cell movements and cell divisions are oriented [1–3] and also the specification of cell fates is influenced by polarity [4–6]. The polarity of individual cells has been studied in *Drosophila* [7,8] and *Caenorhabditis elegans* [9,10], but the establishment and maintenance of polarity in a field of cells is still mysterious [11,12].

It is a general notion that during development of the C. elegans embryo, cell fate specification [13,14] and division orientation [15] are coordinated strictly in the anterior-posterior (a-p) direction. The first cleavage generates an asymmetric 2-cell stage embryo with an anterior AB and a posterior P_1_ blastomere [15]. Most of the cells (70%) of the hatching embryo are derived from the AB blastomere. The first quantitative 4D-microscopic analyses of cleavage directions in the C. elegans embryo showed that AB-derived cells do not cleave strictly along the a-p axis but deviate, on average, approximately 36 ° from the a-p axis until the premorphogenetic stage (256-AB cell stage) of embryogenesis [16]. During early development from the 4-AB to the 64-AB cell stage, the deviation from the a-p axis is even higher (approximately 45 ° ± 20 °; see Figure S1 for examples of cleavages in a normal embryo).

Goldstein reported that in vitro contact with P_2_, the posterior daughter of P_1_, orientates the mitotic spindle in its anterior sister EMS and that P_3_, the posterior daughter of P_2_, does the same to its anterior sister E [17]. Besides the influence of P_1_ descendants on EMS cleavage orientation, it was also shown that the orientation of the ABar division depends on contact with the C blastomere, the anterior daughter of P_2_ [18]. Furthermore, the combination of blastomeres in vitro demonstrated that also individual AB-derived cells of the 12-cell stage embryo align their mitotic spindles towards MS and C [19]; however, the directions of the next cleavages were not reported. Spindle orientation, as well as cell fate specification, was shown to depend on Wnt signalling in C. elegans [19–22].

To investigate cellular polarity in AB-derived cells of the C. elegans embryo, we performed blastomere combination experiments in vitro [23,24]. The generated embryonic fragments were analysed by 4D-microscopy, which allowed us to monitor cell positions, mitoses, movements, and division orientation of all cells during development (see Materials and Methods and [16,25]).

Here we show that P_2_ and its descendants introduce a polar bias into the cleavages of AB-derived cells, which causes a shift of the average division angle with respect to the a-p axis from 62 ° to 45 °. This polarisation depends on a MOM-2/Wnt signal originating from P_2_ and its descendants. Moreover, we provide evidence that P_2_ and its descendants constitute a polarising centre, which signals throughout early development. Furthermore, we show that the Wnt signal is transduced from AB descendant to AB descendant by a relay mechanism.

## Results/Discussion

### Elongation of AB-Derived Embryonic Fragments Depends on the Presence of P_2_

**Figure 1:**
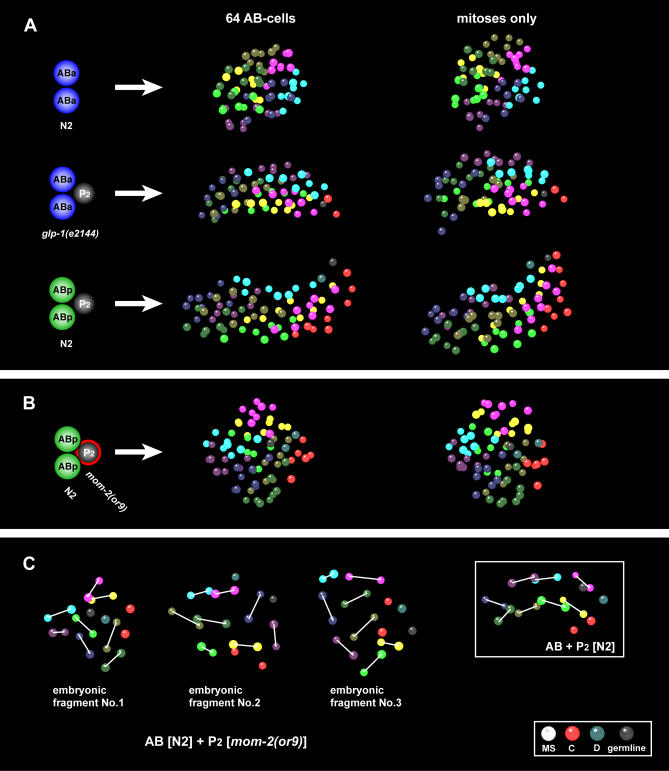
P_2_ Polarises the AB Descendants Depending on MOM-2/Wnt The left column of panels (A) and (B) shows the combination of the early blastomeres used in the experiments. Blastomere genotypes and fates (as determined by lineaging) are indicated. *mom-2* blastomeres are marked with a red margin. The middle column shows the positions of the nuclei at the end of the eighth generation of cells (64 AB descendants) using the 3-D representation feature of SIMI Biocell. Positions were determined by following the development of all cells. AB-derived regions, founded at the 8-AB cell stage (fifth generation) [25], were colour coded independently of their fate to show the topology of each region. P_1_ descendants are colour coded according to their fate. The right column shows 3-D representations of the same generation that illustrate how embryonic fragments look if the contribution of cell movements is removed using an algorithm described in [16] so that only the contribution of mitoses is left (“mitoses only”). In (C) 3-D representations of the sixth generation of cells (16 AB descendants) are shown. To indicate the orientation of the cleavages of their mother cell, the two sisters are connected with a white bar shortly after mitosis. (A) Development of isolated AB blastomeres (the two ABx daughters are shown on the left) compared to AB blastomeres to which a P_2_ blastomere was added after the two daughters were born. P_2_ causes the embryonic fragment to elongate independently of whether only ABa or ABp fates are present. As becomes obvious from the colour code, the AB-derived regions stretch out along the axis defined by P_2_ whereas they do not in isolated AB cells. The elongation is caused by the alignment of mitoses along the a-p axis [see white box in (C) and Figure 2D–2F]. A comparison of the original and the “mitoses only” representations reveals that the shape of the embryonic fragments is mainly due to mitoses. This suggests that a bias in mitosis direction is the cause of the elongation of the embryonic fragments. (B) Chimeric embryonic fragment consisting of a wild-type AB and a *mom-2(or9)* P_2_ blastomere. This *mom-2* allele prevents the elongation of the embryonic fragment completely. Like in an isolated AB blastomere (A), the eight AB-derived regions are not elongated, indicating that the mitoses of all AB-derived cells were not mainly oriented towards the P_2_ blastomere (Figure 2G). Again, the original and the “mitoses only” representations look very similar. (C) Orientation of cell divisions (fifth division of AB-derived blastomeres) in three embryonic fragments where wild-type AB and *mom-2* P_2_ blastomeres were combined [embryonic fragment 1 also shown in (B)] and a wild-type control (white box). In contrast to the wild-type control embryonic fragment, divisions are not directed towards the position of P_2_ and its descendants. For a quantitative analysis of division angles of embryonic fragments, see Figure 2. Furthermore, the shown wild-type embryonic fragment exemplifies that AB-derived cells that do not touch P_2_ also orient their divisions towards P_2_.

**Figure 2:**
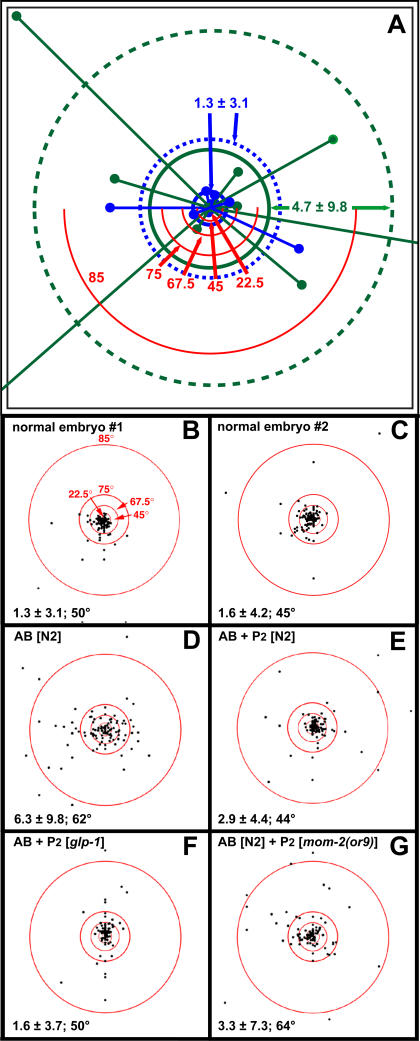
Quantitative Analysis of Cleavage Orientation (A) Scheme explaining the quantitative analysis of cleavage orientation using “Löwe projections”. The directions of cell cleavages of all cells are visualised by dots projected on a target screen in which the centre represents the a-p axis. These dots are obtained in the following way: each dividing cell is placed on the a-p axis and a vector is projected from the centre of the mother cell through the centre of the daughter cell. The point where this vector hits a target screen is marked by a dot (the distance between mother cell and screen along the a-p axis is normalised to 1). The red semi-circles represent particular division angles (as indicated). Embryos were analysed from four to 64 AB-derived cells. For statistical analysis, the mean distance of the dots from the target centre and the standard deviation are calculated. This “Löwe value” and its standard deviation give a reasonable measure of the general direction of cell cleavages. This is exemplified by showing nine cleavages of a “polarised” normal embryo (blue) and a “nonpolarised” isolated AB blastomere (green). The mean is indicated by a circle and the standard deviation by a dotted circle. Note that the representation of cleavage angles is not linear, because the distance from the centre to a dot corresponds to the tangent of the cleavage angle to the a-p axis. Thus, angles close to 90 ° cannot be shown on the target screen (here, this is the case for two cleavages of the “nonpolarised” isolated AB blastomere). It becomes obvious that for the normal embryo, in which cells divide mainly along the a-p axis, most dots are located in close proximity to the centre and the corresponding Löwe value is low. In contrast, the dots are widely scattered for the isolated AB blastomere, in which cells do not mainly divide in the a-p direction, and the Löwe value is high. (B–G) “Löwe projections” of two normal embryos and the embryonic fragments shown in Figure 1A and 1B. The red circles correspond to the red semicircles in (A). In the lower left corner, Löwe values and mean division angles are shown. (B and C) Normal embryos #1 and #2 from [16,25]. Cells mainly divide in an a-p direction. The Löwe value and especially its standard deviation are low. If a third normal embryo (not shown) is also considered, normal embryos have Löwe values between 1.3 and 1.6 with standard deviations ranging from 3.0 to 4.2. The mean cleavage angles vary between 45 ° and 50 °, the standard deviations between 20 ° and 23 °. (D) Isolated AB blastomere. Compared to normal embryos (B and C) and AB blastomeres where a P_2_ blastomere was added (E), the division angles scatter all over the target area; i.e., cells do not mainly divide in the a-p direction. This is reflected in the higher Löwe value and especially the high standard deviation. Five points are off the target screen due to 90 ° divisions. (E) AB blastomere where P_2_ was added. Cells mainly divide in the a-p direction, and the Löwe value and its standard deviation decrease noticeably compared to the isolated AB blastomere in (D). (F) AB blastomere where P_2_ was added in a *glp-1(e2144)* background. Again, cells mainly divide in the a-p direction. (G) Addition of a *mom-2(or9)* P_2_ blastomere to a wild-type AB blastomere. Cells do not mainly divide in the a-p direction. The Löwe value is higher than in embryonic fragments dividing mainly in the a-p direction and its standard deviation resembles the standard deviation of the isolated AB blastomere.

AB blastomeres isolated from P_1_ at the 2-cell stage show an abnormal “spiralian-like” cleavage pattern, in which the direction of cleavages rotates approximately 90 ° after each division [26,27]. This is due to the migration pattern of the centrioles, which always move orthogonally out of the plane defined by the last two divisions [28]. The developing embryonic fragments have a spherical shape (Table 1, row A; Figures 1A and 2D). This suggests that AB descendants do not have any intrinsic polarity to orient their divisions in the normal a-p direction [15,16] and that the a-p polarity of these cells is induced after the 2-cell stage. This appears to disagree with Park and Priess, who showed that sister pairs of 16-AB and 32-AB cell stage AB blastomeres display an asymmetric expression of POP-1, although they never touched P_2_ or its descendants [19]. However, we recently showed that isolated AB blastomeres display a-p asymmetries in cell fates [27]. Thus, the POP-1 asymmetry described by Park and Priess might indicate an asymmetry in cell fates rather than in the polarisation of cleavage direction.

**Table 1:**
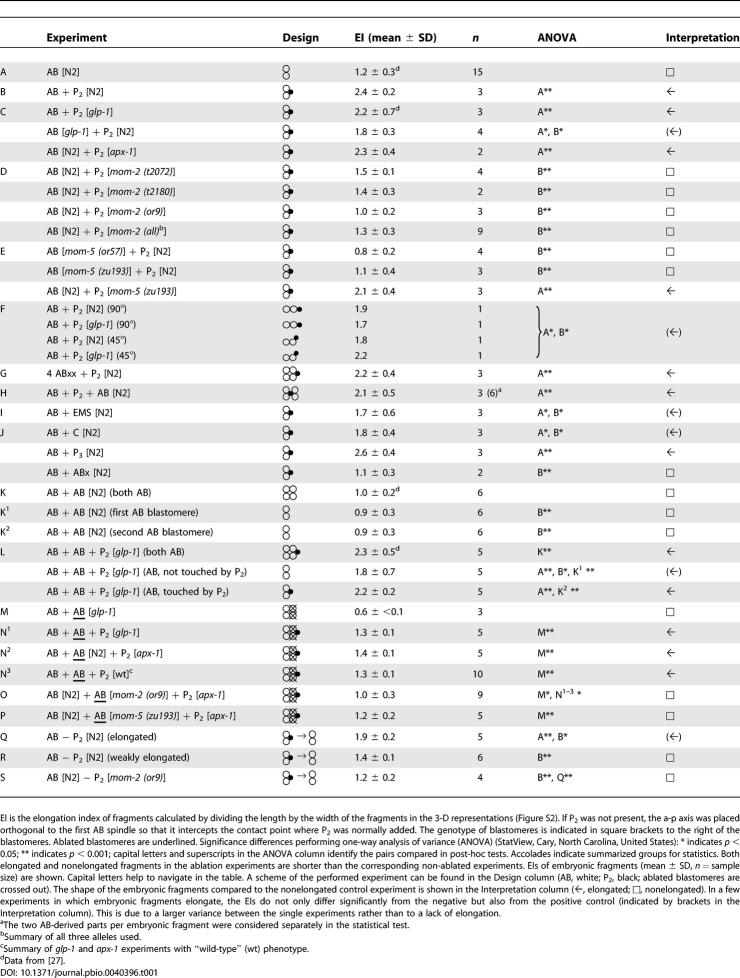
Elongation of Embryonic Fragments

When a P_2_ blastomere is added to the two daughters of isolated AB blastomeres, the developing AB-derived embryonic fragments elongate and become twice as long as they are wide (Table 1, row B; Figure 1A). The eight regions deriving from the eight AB descendants of the 12-cell stage embryo [25] are also elongated. This finding contrasts with the embryonic fragments without P_2_, in which the regions are approximately spherical (Figure 1A). In the elongating embryonic fragments, the cells do not divide “spiralian-like,” but orient towards the added P_2_ blastomere (white box in Figure 1C). This suggests that the observed elongation is due to cleavage orientation.

### Elongation of Embryonic Fragments Results from Oriented Divisions

We then tested whether the orientation of cell divisions was the main cause of the elongation. We used SIMI Biocell to record the 3-D coordinates of the mother cells and then those of the daughters 105 s after the initiation of the cleavage furrow, and we calculated the cleavage angles of all cells relative to the a-p axis (see Materials and Methods). A comparison of division angles between isolated AB blastomeres and AB blastomeres cultured with an added P_2_ blastomere showed a significant difference (*p* < 0.001, Student's *t*-test). In isolated AB blastomeres, average divisions deviated 63 ° ± 20 ° (*n* = 3 embryonic fragments) from the a-p axis, whereas in embryonic fragments with P_2_, divisions deviated on average 45 ° ± 22 ° (*n* = 3) from the a-p axis.

To assess cleavage directions quantitatively, we projected the division angles onto a target screen (Figure 2A) (“Löwe projection”, named after the mathematician Harald Löwe, Braunschweig, who kindly suggested this method). The Löwe value is a measure of the deviation of cleavages from the a-p axis in all three dimensions. An isolated AB-derived fragment demonstrated a Löwe value of 6.3 ± 9.8, which corresponds to the mean angle of 62 ° ± 23 ° with respect to the a-p axis. After addition of P_2_, the Löwe value decreased to 2.9 ± 4.4 and the angle to 44 ° ± 22 ° (Figure 2D and 2E).

The direction of cleavages correlates very well with the variation of shape (elongation) of the embryonic fragments; the statistical correlation between elongation, the average cleavage angle, and the Löwe value are better than 0.92 (*p* < 0.005; considering 2,200 cell divisions). Thus, we used the shape of embryonic fragments to assess division orientation in the large number of embryonic fragments analysed (120 fragments in 30 experiments; Table 1). For many of the experiments, the Löwe projections shown allow a more comprehensive comparison.

To assess the contribution of cell movements between cleavages, we removed these movements from our dataset using an algorithm described in [16]. In this way, only the contribution of mitoses is left and can be seen in the 3-D representations of the developed fragments (Figure 1). Even after this procedure, embryonic fragments including P_2_ were approximately two times longer than those derived from only AB blastomeres (Figure 1A). Thus, the shape of the embryonic fragments depends almost entirely on the cleavage directions of cells. Our measurements show that the addition of P_2_ to an AB blastomere causes a significant alteration of cleavage orientation of AB-derived cells of the embryonic fragment which, in turn, leads to an elongation.

The analyses also indicate that the alteration of cleavage direction is not limited to the AB descendants that touch P_2_, but occurs in all AB descendants (white box in Figure 1C). P_2_ biases division orientation from a “spiralian-like” pattern observed in isolated AB blastomeres (corresponding to an average angle of approximately 60 °; i.e., the mean of three cleavages with angles of 0 °, 90 °, and 90 ° with respect to the a-p axis) to 45 ° ± 22 °. This result is very similar to the 45 ° ± 20 ° (n = 3) observed for the cleavages in early development of normal embryos (Figure 2B and 2C).

At first sight, our results differ from studies in C. elegans that have shown that isolated AB-derived blastomeres of the 12-cell stage embryo [19] and the EMS blastomere [17] divide directly towards an added P_2_ blastomere (0 °) in their first division [22]. However, in these studies, the behaviour of cells in subsequent divisions was not discussed. When we analysed division orientation in normal embryos between the 4-AB and the 64-AB cell stage (the first two divisions of AB occurring perpendicular to the a-p axis), we observed that AB-derived cells divide rarely 0 ° but on average 45 ° ± 20 ° with respect to the a-p axis (e.g., Figure S1 and Figure 2B and 2C). Also, in vivo (in contrast to in vitro [17])*,* EMS does not divide directly towards P_2_. In the five normal embryos we analysed, the divisions occurred on average 35 ° ± 6 ° off the a-p axis. Thus, our observations in embryonic fragments are consistent with normal embryonic development.

In normal development, an average cleavage angle of 45 ° may reflect the optimal compromise between minimising the need for compensatory cell movements to less than one embryo length per cell [16] while still allowing a stringent a-p assignment of cell fates [15]. Strict a-p cleavages would displace cells 40 times the embryo length in eight cleavages (see Protocol S1 for further discussion).

### Cleavage Angles Are Independent of Cell Fate

Because the addition of P_2_ induces an ABp fate in the daughters of AB [29], and because ABp-derived regions are longer than those derived from ABa [25], the elongation of embryonic fragments could merely reflect the presence of ABp-derived fates; i.e., the bias in division orientation might only be due to the execution of specific cell fates. We therefore prevented the ABp induction by using different combinations of blastomeres in which either the responsible receptor GLP-1/Notch or its ligand APX-1/Delta were mutant [29], and we then analysed the elongation of these embryonic fragments. Below, we refer to blastomeres from embryos whose mothers were homozygous for a mutation, for example in *glp-1,* as *glp-1* blastomeres. When *glp-1* AB and P_2_ blastomeres are combined, cells mainly divide away from P_2_ and the embryonic fragments elongate (Table 1, row C; Figures 1A and 2F). The same is true when other blastomere combinations are used that also prevent the induction of ABp (Table 1, row C). Thus, the induction of ABp-derived fates by P_2_ is not the reason for the elongation of the fragments. Based on these results, we instead suggest that P_2_ and/or its descendants influence division orientation by being the source of a polarising signal.

### Orientation of Cleavages Depends on a Wnt Signal Originating from P_2_

Earlier studies have shown that MOM-2/Wnt is involved in spindle orientation in EMS and in individual AB descendants [19–22]. To test if the polarisation of embryonic fragments depends on the MOM-2/Wnt ligand, we generated chimeric fragments by combining wild-type AB and *mom-2* P_2_ blastomeres. Three different mutant alleles interfere with the elongation of the fragments (Table 1, row D). Fragments in which P_2_ was derived from *mom-2(or9)* do not elongate at all, because the divisions of the AB-derived blastomeres and their descendants were not aligned towards P_2_ (Figures 1B, 1C, and 2G). These results suggest that MOM-2/Wnt is the relevant signal given out by P_2_ or its descendants. The polarisation also depends on the MOM-5/Frizzled receptor [20,21] in AB but not in P_2_ (Table 1, row E). That MOM-2 and MOM-5 organise division orientation can also be seen in mutant embryos. Cell divisions in AB descendants between the 4- and 64-AB cell stage deviate from the a-p axis by 55 ° ± 19 ° in *mom-2(or9)* embryos and by 55 ° ± 20 ° in *mom-5(zu193)* embryos; this is significantly different from wild-type embryos (40 ° ± 20 ° mean deviation from the a-p axis, embryos #2 and IB+RS [16]; Student's *t*-tests: *p* < 0.001 [*n* = 2 each]).

### A Posterior Polarising Centre

By analysing the polarising signal of P_2_ and its descendants in detail, we found that these cells display properties of a polarising centre. Regardless of whether P_2_ is added in a 45 ° or a 90 ° angle (as compared with the normal experimental conformation), P_2_ always defines the posterior pole of the fragment and induces an elongation (Table 1, row F). Also, embryonic fragments consisting of P_2_ and the granddaughters of AB (instead of the daughters of AB) elongate, suggesting that polarisation does not depend on the establishment of contact between blastomeres at a particular developmental stage (Table 1, row G). The signal of P_2_ acts in all directions, because the addition of two AB blastomeres to one P_2_ blastomere on opposite sides leads to an elongation of both AB-derived parts (Table 1, row H). In an embryonic fragment consisting of EMS, AB, and P_2_, the descendants of AB and EMS orient towards the descendants of P_2_, showing that P_2_ and its descendants are dominant over the P_2_ sister EMS and its descendants (Figure S3A). However, in the absence of P_2_, the EMS blastomere has a polarising activity itself (Table 1, row I) which was reported before by Park and Priess [19]. The addition of two P_2_ blastomeres to the daughters of one or two AB blastomeres from opposing directions leads to two elongated structures with opposing polarities (Figure S3B). Adding one of the daughters of P_2_ (either C or P_3_) to the two AB daughters causes an elongation of the embryonic fragment, whereas the addition of another AB daughter (which never touched P_2_) does not (Table 1, row J). This shows that the two daughters of P_2_ are able to polarise AB-derived embryonic fragments and that this effect is specific to P_2_ and its descendants. This supports Park and Priess, who showed the same for single AB-derived blastomeres [19]. Taken together, these results show that in all combinations tested, P_2_ and its descendants define the posterior pole of an elongated structure and orient the cell divisions of AB-derived blastomeres.

**Figure 3:**
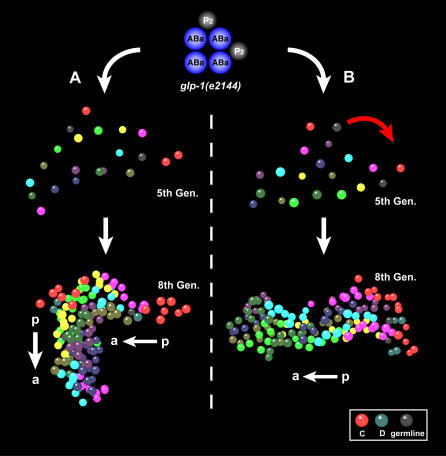
P_2_ and Its Descendants Organise Polarity Continuously For details, see legend to Figure 1. Two mutually perpendicular P_2_ blastomeres were added to the daughters of AB in a *glp-1(e2144)* background. Two different outcomes of the experiment are shown in (A) and (B). (A) All the P_2_ descendants stay in their original position; an L-shaped pattern with two a-p axes is formed. (B) The descendants of one P_2_ blastomere shifted posteriorly and joined the other P_2_ descendants (red arrow). This displacement of the P_2_ descendants began in the shown fifth generation. It resulted in an elongated structure with only one a-p axis as can be seen in the eighth generation.

The following experiments also argue that P_2_ and its descendants constitute a polarising centre. When two mutually perpendicular P_2_ blastomeres were added to four AB daughters, two different patterns developed from the same starting configuration (Figure 3). Because in both experiments *glp-1(e2144)* blastomeres were used, any influence of different cell fates can be excluded. In one fragment, the descendants of the two P_2_ blastomeres remained in their initial positions, resulting in an L-shaped structure with two a-p axes. In the other fragment, the descendants of one P_2_ remained in their original position for 75 min, until after the P_3_ division, and then moved and joined the descendants of the second P_2_. This resulted in an elongated structure with only one a-p axis. This finding indicates that the AB-derived blastomeres respond continuously to a polarising signal; they even reorganise their polar behaviour when the P_2_ descendants move. This experiment again demonstrates that the descendants of P_2_ provide the polarity signal, because not P_2_ itself but only its descendants reached the posterior part of the embryonic fragment and caused its elongation. An experimental displacement of cells, recorded under the 4D-microscope while development is progressing appears to be impossible to us at this time. Thus, the coincidentally occurring movement of the P_2_ descendants observed here was a unique chance to examine the consequences of the displacement of signalling cells during development.

### The Polarising Signal Reaches Cells Not Touching P_2_

Some of the experimental results imply that cells that are not directly touched by P_2_ or its descendants are also affected by the polarising signal. To test this, we combined two pairs of daughters of AB blastomeres with one P_2_ blastomere so that only one pair of AB daughters touched P_2_. To ensure that P_2_ did not induce the ABp fate in the touched AB-derived blastomeres, *glp-1* blastomeres were used. The descendants of both AB blastomeres form elongated structures, in contrast to the controls without P_2_ (Table 1, rows K and L). Thus, the polarising signal is transported to AB-derived blastomeres not touching P_2_.

### How Is the Polarising Signal Transported?

**Figure 4:**
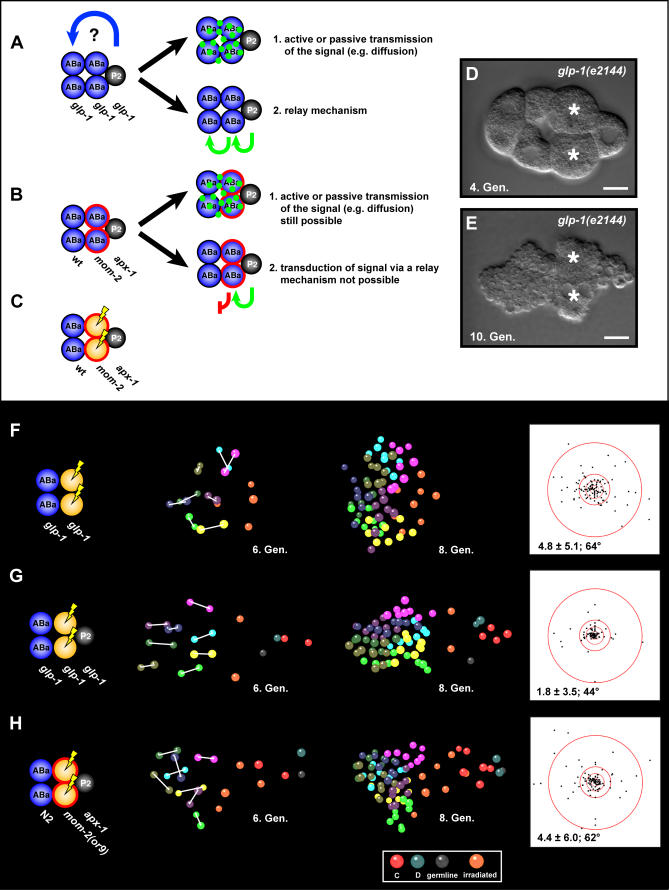
The Polarising Signal Is Transferred from AB Blastomere to AB Blastomere by a Relay Mechanism For details see legend to Figure 1. *mom-2* blastomeres are marked by a red outline. (A–C) Schemes showing the designs of experiments. (D and E) DIC images of the fragment analysed in (G). Bars, 10 μm. (D) shows fragment after laser ablation of the two central blastomeres. Irradiated cells are marked with asterisks. (E) shows terminal stage of the fragment. (F–H) Experiments where the central AB descendants were irradiated. The leftmost column shows the designs of the experiments. In the next column, division angles of the 16-AB cell stages are indicated by a white bar connecting sister cells shortly after mitosis. Further to the right, the 3-D representations of the 64-AB cell stages are shown. The column most to the right shows the Löwe projections of the division angles. (F) and (G) show two of the control experiments using wild-type, *glp-1,* or *apx-1* blastomeres (Table 1, rows M and N). (F) Control experiment without P_2_. No elongation of the nonablated part of the AB-derived embryonic fragment occurred. (G) Control experiment with P_2_ *glp-1* background to prevent induction of ABp fates. The mitosis directions of the fifth division, which produces the sixth generation of cells, in the nonablated part of the AB-derived embryonic fragment indicate a polarisation of the blastomeres. The fragment elongates. This experiment shows that laser ablation does not hinder a potentially diffusing or transported signal. (H) Two *mom-2* AB-derived daughters between P_2_ and wild-type AB-derived cells prevent elongation (Table 1, row O). Mitoses are not directed towards P_2_.

It is not clear how signalling molecules are transported [30]. A secreted molecule could be transmitted actively, it could spread passively by diffusion, or it could be passed on from cell to cell by relay. In a relay mechanism, it would be produced newly in each cell in response to the signal from the neighbouring cell [31,32] (Figure 4A). To discriminate among these possibilities, we put a *mom-2* AB blastomere between a wild-type AB and an *apx-1* P_2_ blastomere. If the signal were transduced via a relay mechanism, the *mom-2* blastomere would inhibit further signalling, because it could not produce a MOM-2/Wnt dependent signal. In contrast, an actively or passively transported signal should still reach the AB descendants (Figure 4B). In these experiments, *apx-1* or *glp-1* blastomeres were again used to obtain only ABa-derived fates. To prevent cells of the different genotypes from intermingling at a later stage, we laser irradiated the two *mom-2* AB daughters after 10 min to prevent further divisions. The cells were not killed (Figure 4D and 4E). Control experiments showed that laser ablation did not hinder the transmission of the polarity signal (Table 1, rows M and N; Figure 4F and 4G). We found that the wild-type AB-derived parts of the fragments, which never touched P_2_, did not elongate when a *mom-2* AB blastomere was placed between a wild-type AB and an *apx-1* P_2_ blastomere (Table 1, row O; Figure 4H). Moreover, the directions of mitoses deviated strongly from the axis defined by P_2_, indicating that the cells were not polarised. The Löwe projections of the cleavage angles for this experiment and the controls are shown in Figure 4F–4H. These experiments suggest that the polarising signal is relayed from cell to cell in C. elegans. The MOM-2/Wnt signal is not far-ranging but must be produced anew as each neighbouring cell receives the signal; this is not possible in the *mom-2* mutant blastomere.

Placing a *mom-5* AB blastomere between a wild-type AB and an *apx-1* P_2_ blastomere also impairs the elongation of the wild-type part of the embryonic fragment (Table 1, row P). Embryonic fragments elongate less as compared with wild-type controls; however, the effect is not as extensive as in experiments using *mom-2* blastomeres. These findings confirm that MOM-5 is needed in AB blastomeres to receive the MOM-2/Wnt signal.

### Polarised AB-Derived Blastomeres Can Polarise Nonpolarised AB-Derived Blastomeres

**Figure 5:**
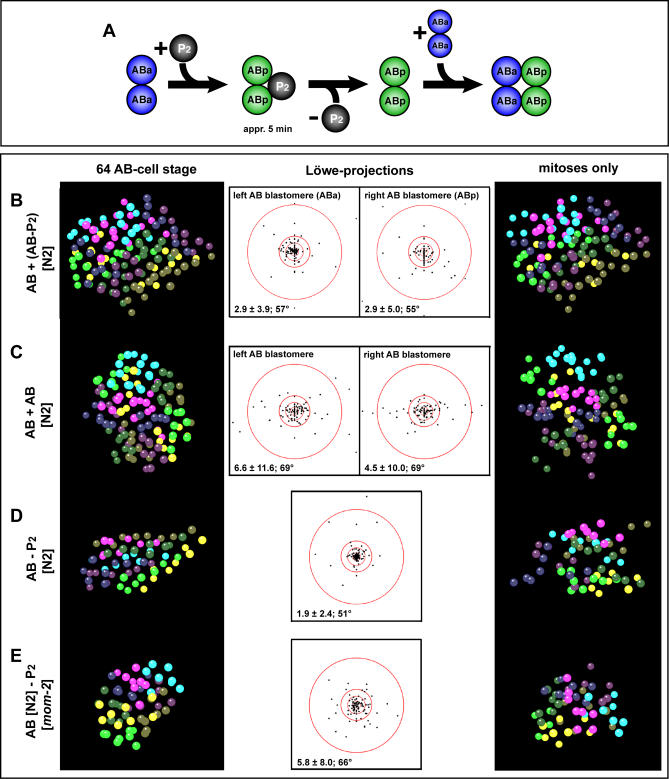
Polarised AB Descendants Transfer Their Polarity (A) Design of the experiment shown in (B). (B–E) Left column: 3-D representations of the 64-AB cell stages of various experiments. The eight AB-derived regions are colour-coded independently of their fate to show the topology of each region. Middle column: “Löwe projections” (see Figure 2A for explanation). Right column: 3-D representations from which the contribution of cell movements was removed (“mitoses only”, see legend to Figure 1). In all experiments presented, these “mitoses only” representations resemble the original embryonic fragment, which suggests that the shape of the embryonic fragments is mainly due to cleavage orientation. (B) Combination of an isolated AB blastomere (ABa) and a blastomere which touched P_2_ transiently (ABp). Four of the eight AB-derived regions of the ABp blastomere (right side of embryonic fragment; blue, violet, dark yellow, and light yellow) elongate comparable to regions in the experiment where a single isolated AB transiently touched P_2_ (see Figure 5D). These regions align in a V-shape configuration, possibly because their elongation is hampered by the descendants of the other AB blastomere (left side of embryonic fragment). The “Löwe projections” show that the cells of the ABp blastomere (right) divide mainly in the direction of the point where P_2_ was transiently added. Additionally, the cleavages of the originally nonpolarised ABa blastomere (left) are mainly directed towards the added polarised ABp blastomere (which is here represented by the centre of the target area). (C) Combination of two isolated AB blastomeres. Both cells have never been exposed to a polarising signal, similar to the ABa blastomere in (B). A round structure with nonelongated regions forms (Table 1, row K). The direction of cleavages appears to be nonpolarised compared to (B). (D) Single AB blastomere that transiently touched P_2_. Cells mainly divide in the direction where the point of contact between AB and P_2_ has been. A nicely elongated structure forms (Table 1, row Q). This elongation resembles the behaviour of the polarised ABp blastomere in (B). (E) Experiment in which a *mom-2* P_2_ blastomere touched a wild-type AB blastomere transiently. The ABp fate is induced by P_2_ but, because of the absence of a polarising signal, cells behave in a nonpolarised way compared to the experiment where a wild-type P_2_ blastomere was added transiently to AB-derived blastomeres (D).

We then sought further evidence that polarised AB descendants are indeed able to produce a polarising signal themselves. To show that the polarisation of neighbours is independent of the original source (i.e., P_2_ or its descendants), we conducted the following technically difficult experiment (Figure 5A). We added the two daughters of an AB blastomere to P_2_. After 5 min, we removed P_2_ and added the now polarised AB blastomeres to freshly isolated AB daughters. The cleavages of these originally nonpolarised blastomeres were not nondirected as in an isolated AB blastomere, but were in many cases directed towards the polarised AB descendants (Figure 5B). The descendants of the AB blastomere that was transiently in contact with P_2_ show elongated AB-derived regions. This experiment demonstrates that AB-derived blastomeres that transiently touch P_2_ become polarised and, furthermore, are able to transduce their acquired polarity to other nonpolarised AB-derived cells.

### A Transient Contact of P_2_ Is Sufficient to Polarise AB-Derived Blastomeres

To further corroborate the finding that AB-derived blastomeres can be polarised by transient contact of P_2_, we tested the influence of P_2_ on single AB blastomeres. The behaviour of single AB-derived blastomeres is not different from two combined AB-derived blastomeres; in both, cells do not divide mainly in an a-p direction and the fragments have rounded shapes (Figure 1A, 2D, and 5C; Table 1, rows A and K).

First, we analysed isolated AB blastomeres that touched wild-type P_2_ transiently (Figure 5D). In five of 12 experiments, the AB-derived fragment elongated (Table 1, row Q); in six fragments, it elongated weakly (Table 1, row R), and in one fragment, no elongation was observed. Second, we analysed AB blastomeres that transiently touched *mom-2* mutant P_2_ blastomeres, and we found that in all four cases, the resulting embryonic fragments did not elongate (Figure 5E; Table 1, row S). These experiments confirm the finding above that a transient signal of P_2_ can polarise AB-derived blastomeres. Furthermore, the latter experiment again suggests that MOM-2 is the polarising signal. It also shows that an induction of ABp fate by P_2_ is not the reason for an elongation of the embryonic fragment, because the induction of ABp occurs also in *mom-2(or9)* mutant embryos (unpublished data).

### Establishment and Maintenance of Polarity in the C. elegans Embryo

Our investigations argue that the a-p polarity of AB descendants in C. elegans is not an autonomous feature established early in embryonic development, but rather that this polarity is induced in AB descendants by P_2_ for the first time at the 4-cell stage. We show that the division orientations of most of the AB descendants are biased towards P_2_ and its descendants (Figures 2 and 5) and that AB-derived cells continuously orient their divisions towards it, even when these signalling cells move during development (Figure 3). This suggests that P_2_ and its descendants constitute a polarising centre in the posterior of the embryo. We further show that MOM-2/Wnt—which has already been implicated in certain aspects of polarity of the early C. elegans embryo [19–22]—is the polarising signal (Figure 1 and 2; Table 1, row D). Moreover, we demonstrate that the MOM-2/Wnt signal is transduced from AB blastomere to AB blastomere by a saltatory relay mechanism (Figure 4).

We restricted our experiments to the AB-derived part of the embryo. An interesting question is how polarity is established and maintained in the remainder of the embryo derived from P_1_. To address this question, we compared the cleavage angles in wild-type and *mom-2(or9)* embryos starting at the 8-cell stage embryo for the next four cleavage rounds. The P_1_-derived cells from MS, E, C, and D cleave with an average angle of 35 ° ± 21 ° in the wild-type embryos as compared to 57 ° ± 21 ° in the *mom-2* embryos. Thus, a lack of MOM-2 affects the cleavage orientations in the P_1_-derived cells as significantly as in the AB-derived cells (Student's *t*-tests: *p* < 0.001 [*n* = 2 each]). It may therefore be that the cleavages of all cells of the embryo are oriented by the same mechanism. Then the polarising activity of MS and C [19] would not be an autonomous feature of these blastomeres but would reflect a polarisation they acquired directly from the P_2_ daughter, P_3_ (in the case of C), or indirectly through a relay mechanism (in the case of MS).

Taken together, our findings might explain how polarity in the C. elegans embryo is maintained during ongoing development; after the identity of P_2_ has been specified by the *par* genes [33]—based on the initial polarisation of the embryo during fertilisation [34]—P_2_ might induce polarity in its neighbours ABp and EMS via a MOM-2/Wnt signal through direct contact [19,22]. Afterwards, polarity of all AB descendants might be continuously maintained by the posterior polarising centre whereas the saltatory relay mechanism would ensure that the polarity signal reached all AB-derived cells.

Because there is emerging evidence that the MOM-2/Wnt signalling pathway in the C. elegans embryo shows some similarity to the planar cell polarity pathway [19,22,35], our findings may provide a paradigm of how polarity can be organised in a field of cells in other organisms, such as planar cell polarity in *Drosophila* [11,12] or convergent extension [6] and gastrulation [3] in vertebrates.

## Materials and Methods

### Strains and genetics.

Worms were grown at 25 °C as described [36]. The following strains and mutations were used: N2 Bristol [36]. LG I: *mom-5(zu193)* [20]; *mom-5(or57)* [21]; *unc-13(e1091)* [37]; *dpy-5(e61)*. LG III: *glp-1(e2144)* [38]. LG V: *mom-2(t2180)* (unpublished data); *mom-2(t2072)* (unpublished data); *mom-2(or9)* [21]; *unc-24(e138), dpy-11(e224), him-9(e1487); apx-1(t2063)* [16]; *apx-1(hd87)* (unpublished data). Unless noted otherwise, mutations were previously described in [39].

### In vitro culture of blastomeres.

The in vitro culture of embryos was described previously [27]. Embryos were cultured in embryonic growth medium supplemented with egg yolk [23]. Unless noted otherwise, a P_2_ blastomere was added to two ABx blastomeres (derived from one AB blastomere divided once) in a way that both ABx blastomeres were touched by it. Two ABx blastomeres were used to ensure that cells of the same embryonic stage were added to P_2_.

### 4-D microscopy.

Methods for 4D-microscopy were described previously [25]. Modifications of the 4D-microscope system are described in [16]. Embryos were recorded at 25 °C.

### Lineage analysis.

The 4D-recordings were analysed using the database SIMI Biocell (SIMI Reality Motion Systems, Unterschleissheim, Germany; http://www.simi.com) [16,25]. Cells were followed and their 3-D coordinates were saved on average every 2 min. Cell cleavages were assessed by marking the position of the mother cell immediately before the cleavage furrow ingresses and the position of its two daughters three frames (105 s) later. By following every cell in the recording, the complete cell lineage of an embryo or an embryonic fragment is built. We only show 3-D representations of the eighth generation (64 AB descendants), because the elongation can be observed best here. When necessary, fates were determined as described in [27]—e.g., to assess if an induction of the ABp fate by P_2_ occurred or not.

### Calculation of division angles.

The software for the quantitative analysis of cleavage orientations was written in Borland Delphi (Borland Software Corporation, Cupertino, California, United States). Division angles relative to the a-p axis were calculated using the straight line defined by the 3-D coordinates of the mother cell and its posterior daughter 105 s after the cytokinesis furrow starts to ingress. Angles of all cleavages from the 4- to the 64-AB cell stage were assessed. For the definition of the a-p axis in embryonic fragments, see Figure S2. Divisions were always described using angles from 0 ° to 90 °, which allows us to use linear statistics with our data.

## Supporting Information

Figure S1 Mitoses May Deviate Significantly from the a-p Axis in Normal Embryos 3-D representation of a 87-cell stage embryo (64 AB descendants, embryo IB + RS; [16]). Anterior is to the left. Colours refer to cell fate as indicated at the bottom. The cell movements (white bars) and mitoses (arrows) of the precursors of four cells (ABarpaap, ABalpapp, ABpraapp, and ABprppppp) from the 8-cell stage (four AB descendants) onwards are projected into the 3-D representation. Green arrows show anterior divisions; red arrows show divisions which are opposed to the overall movement of the cell. Mitoses are not strictly oriented in a-p direction but may deviate strongly from the a-p axis as shown in these examples: ABarpaap 45 ° ± 23 °, ABalpapp 49 ° ± 8 °, ABpraapp 52 ° ± 26 °, and ABprppppp 61 ° ± 25 °. The average mitosis angle of all AB descendants in this embryo is 41 ° ± 20 ° with respect to the a-p axis from the 8-AB to the 64-AB cell stage.
(1.8 MB TIF)

Figure S2 Quantitative Analyses The elongation index is calculated by dividing the length of the fragment (red line) by the width of the fragment (green line) in the 3-D representations at the 64-AB cell stage (eighth generation). The a-p axis is defined by P_2_ and the AB-derived blastomere, which is placed farthest from P_2_. This axis is always assessed at the time point at which the measurement is taken (at the 64-AB cell stage), because P_2_ and its descendants move during development (due to their and their neighbours' divisions) and cells orient continuously towards P_2_ and its descendants.
(766 KB TIF)

Figure S3 Development of Embryonic Fragments in which Two P_2_ Descendants Were Added to AB-Derived Blastomeres (A) P_2_ overcomes the polarising activity of EMS descendants. The regions are oriented only towards P_2_ (Table 1, row I). The EMS descendants move from an anterior to a lateral position during development (bent arrow).
(B) Two P_2_ blastomeres, which were added to the two AB daughters from opposite sites, induce two elongated structures with opposing polarity (white arrows). If the number of cells is increased by using two AB blastomeres, then again two elongated structures form (white arrows). However, the cells which are further away from the P_2_ blastomeres loop out to form one common axis (red arrow) which indicates, as discussed in the main text, that cells permanently interact to define the a-p axis.
For details see legend to Figure 1.
(1.1 MB TIF)

Protocol S1 Supplemental Discussion (25 KB DOC)

## Abbreviations

a-p: anterior-posterior
