# Supplementary material for: A Posterior Centre Establishes and Maintains Polarity of the Caenorhabditis elegans Embryo by a Wnt-Dependent Relay Mechanism
Source: PLoS Biol. 2006 Nov 21;4(12):e396. doi: 10.1371/journal.pbio.0040396 (PMC1637133; doi:10.1371/journal.pbio.0040396)
Supplement: Supplementary file 1 [file pbio.0040396.sd001.doc]

**Protocol S1**

**Supplemental Discussion**

Regulation of cleavage direction in normal and manipulated development

Sulston et al. (1983) generally assigned a-p directions to cell cleavages in the embryo [15]. Schnabel et al. (2006) evaluated cleavage directions quantitatively in three wild-type embryos and came to the conclusion that the cleavages deviate on average 36° from the a-p axis between the 8-AB and the 256-AB cell stage [16]. Cleavage directions of specific cells vary from embryo to embryo. During early development (4 to 64-AB cell stage), cleavages deviate on average 45° from the a-p axis and the standard deviations are between 20° and 23°. Thus, many of the cleavages are more directed towards the lateral axes (l-r and/or d-v) of the embryo than to the a-p axis. The assignment of cleavages in a-p direction may be justified for the sake of simplicity but it does not reflect the true situation in the embryo [16]. An observer watching the divisions live under the microscope, however, has no alternative to that approach.

It is an interesting question why cells are not directed more precisely in an a-p direction. We think that a more precise aiming in a-p direction would be counterproductive since cells would then be transported very far during embryogenesis and would have to migrate long distances back to compensate for that. When a cell divides into two daughters of equal size only in one (a-p) direction, the distance cells are passively moved increases by a factor of 1.6 per generation. The diameter of a daughter is defined by the third root of 0.5 (the volume is half compared to the mother), which is 0.794. Considering that cells become smaller each generation but also double in number, they would move 6.35 times of the diameter of the first cell in four generations and 40.4 times of the diameter of the first cell in eight generations. Thus, each descendant of AB would have to migrate on average approximately 9.5 embryo lengths to compensate for this displacement. However, if division angles rotate by 45° relative to the a-p axis the passive transport of cells is reduced to 2 embryo lengths. If the angles additionally rotate around the l-r and d-v axes by 45° the cell movements needed to compensate for the passive transport during the cleavages should be reduced to less than 0.25 embryo length. Thus, it appears that a deviation of 45° from the a-p axis is an optimal value to minimise the displacement of cells during embryogenesis.

In our experiments, we generally observe that the addition of the polarising P2 blastomere restricts the cleavage directions of the AB-derived blastomeres from a value higher than 60° to less than 50° relative to the a-p axis. Thus, the polarity signal reduces the tumbling of the spindles – the standard deviation is reduced by a factor of two. An average effect of approximately 10° reduction may appear to be rather low, yet it increases the transport on the a-p axis by a factor of 1.14 per cleavage. This accumulates to an increase of length by a factor of 1.7 in four generations. This corresponds to the elongation indices we observe after an addition of P2 to AB if only mitoses are considered. The additional effect of the observed elongation (about 2) is explained by the packing of cells, which occurs after the divisions.
